# Current concepts in acromioclavicular joint (AC) instability – a proposed treatment algorithm for acute and chronic AC-joint surgery

**DOI:** 10.1186/s12891-022-05935-0

**Published:** 2022-12-09

**Authors:** Daniel P. Berthold, Lukas N. Muench, Felix Dyrna, Augustus D. Mazzocca, Patrick Garvin, Andreas Voss, Bastian Scheiderer, Sebastian Siebenlist, Andreas B. Imhoff, Knut Beitzel

**Affiliations:** 1 Department of Orthopaedic Sports Medicine, Technical University of Munich, Ismaninger Str. 22, 81675 Munich, Germany; 2 Department of Orthopaedics and Trauma Surgery, Musculoskeletal University Center Munich (MUM), University Hospital, LMU Munich, Munich, Germany; 3 Department of Trauma, Hand and Reconstructive Surgery University Hospital Münster, Munich, Germany; 4 Massachusetts General Hospital, 55 Fruit St, Boston, MA 02114 USA; 5 Department of Trauma Surgery, University Regensburg, Regensburg, Germany; 6 Arthroscopy and Orthopedic Sportsmedicine, ATOS Orthoparc Clinic, Cologne, Germany

**Keywords:** Acromioclavicular joint, Acromioclavicular joint reconstruction, AC joint, Horizontal instability, Vertical instability, Anatomic acromioclavicular joint reconstruction

## Abstract

**Background:**

There exists a vast number of surgical treatment options for acromioclavicular (AC) joint injuries, and the current literature has yet to determine an equivocally superior treatment. AC joint repair has a long history and dates back to the beginning of the twentieth century.

**Main body:**

Since then, over 150 different techniques have been described, covering open and closed techniques. Low grade injuries such as Type I-II according to the modified Rockwood classification should be treated conservatively, while high-grade injuries (types IV-VI) may be indicated for operative treatment. However, controversy exists if operative treatment is superior to nonoperative treatment, especially in grade III injuries, as functional impairment due to scapular dyskinesia or chronic pain remains concerning following non-operative treatment. Patients with a stable AC joint without overriding of the clavicle and without significant scapular dysfunction (Type IIIA) may benefit from non-interventional approaches, in contrast to patients with overriding of the clavicle and therapy-resistant scapular dysfunction (Type IIIB). If these patients are considered non-responders to a conservative approach, an anatomic AC joint reconstruction using a hybrid technique should be considered. In chronic AC joint injuries, surgery is indicated after failed nonoperative treatment of 3 to 6 months. Anatomic AC joint reconstruction techniques along with biologic augmentation (e.g. Hybrid techniques, suture fixation) should be considered for chronic high-grade instabilities, accounting for the lack of intrinsic healing and scar-forming potential of the ligamentous tissue in the chronic setting. However, complication and clinical failure rates remain high, which may be a result of technical failures or persistent horizontal and rotational instability.

**Conclusion:**

Future research should focus on addressing horizontal and rotational instability, to restore native physiological and biomechanical properties of the AC joint.

## Background

Current literature covers a vast number of surgical treatments for acromioclavicular (AC) joint injuries, emphasizing the incertitude regarding the best treatment. Historically, a few of these techniques have been abandoned or re-invented secondary to high complication and clinical failure rates. Classically, the Rockwood Classification attempts to characterize these injuries based upon the associated ligamentous injury and degree of displacement. This is highlighted in the Table 1. Current concepts reviews, systematic reviews and meta-analyses have attempted to reveal the evidence for the optimal treatment of AC joint injuries [1–6].

**Table 1:** Rockwood Classification of Acromioclavicular Joint Injury

Type	Definition	Physical Examination	Radiographic features
I	• AC ligament sprain with ligaments intact • No displacement or instability	• Tenderness at ACJ • Provocative tests positive (cross body adduction, etc)	• No obvious radiographic abnormality
II	• Ac ligaments torn • CC ligaments sprained but intact	• ACJ subluxation/displacement with provocative stress	• Can show subtle distal clavicle elevation, but no obvious separation
III	• Disruption of AC and CC ligaments • A/P and superior/inferior instability	• Superior displacement of the distal clavicle • Acromion and shoulder girdle inferior to distal clavicle	• Radiographs may show up to 100% or greater increase in CC distance
IV	• Disruption of AC and CC ligaments • Posterior displacement of distal clavicle through trapezius	• Palpable distal clavicle posteriorly through trapezial fascia • Also associated with anterior SC joint injury/dislocation	• Subtle findings on AP/Zanca view • Critical to evaluate for posterior distal clavicle displacament on axillary view
V	• Disruption of AC and CC ligaments • Significant displacement of acromion/scapula due to weight of the extremity	• Gross superior displacement of distal clavicle and inferior translation of acromion/scapular complex • Can protrude through trapezial/deltoid fascia and tent skin	• CC distance grossly increased, greater than 100%
VI	• Inferior displacement/dislocation of distal clavicle • Can be displaced subacromial or subcorocoid	• Can palpable defect and displaced distal clavicle • Neurovascular exam critical to rule out associated neurovascular compromise	• Detect distal clavicle in subcorocoid/subacromial position

Operative treatment may be reserved for heavy physical laborers, younger patients, overhead athletes, and frequent overhead users [7–10]. However, little evidence supports the claim that these patients would significantly benefit from operative treatment when compared to nonoperative treatment [11]. Especially, long-term data comparing operative versus nonoperative treatment is limited [4–6, 12].

AC joint repair has a long history and dates back to 1917, when Cadenet first introduced his technique for AC joint instabilities [13]. Since then, over 150 different techniques have been described [14], covering open and closed techniques including metallic wires, pins, hook plates, auto- or allografts, suspension devices, synthetic ligaments, ligament or tendon transfers, clavicle osteotomy or excision. Complication rates have been reported to vary widely between 5 and 30% of cases, mostly depending on the type of repair [15].

Currently proposed surgical methods vary widely. These include anatomic coracoclavicular ligament reconstruction, coracoacromial ligament transfer, reconstruction with internal fixation, and reconstruction with implantable suture fixation devices. This review of current concepts aims to give an overview of the relevant biomechanics and pathoanatomy, review the current available treatment modalities, and highlights current challenges while pointing out the authors’ preferred treatment algorithm.

## Main text

### Epidemiology and Pathoanatomy

The highest prevalence of AC joint injuries have been reported in 20- to 30- year old male patients participating in high contact sports, with AC joint injuries generally accounting for 12% of all shoulder injuries in the overall population [16]. The mechanism of most AC joint injuries is a direct fall on the superolateral aspect of the shoulder with the arm in an adducted position. In contrast, indirect injury occurs by falling on the outstretched arm, causing the humeral head to translocate superiorly and drive the humeral head into the acromion.

### Acromioclavicular anatomy and biomechanics

The acromioclavicular joint is formed from the diarthrodial articulation between the distal end of the clavicle and the acromion process of the scapula, and is stabilized by various static and dynamic constraints. The acromioclavicular (AC) ligament complex is comprised of anterior,posterior, superior, and inferior ligaments. The corococlavicular (CC) ligaments are composed of the trapezoid and conoid ligaments. These AC and CC ligamentous complexes are the static stabilizing structures of the AC joint. The dynamic stabilizers include the trapezius and deltoid musculofascial attachments [1, 17–19].

The AC ligament complex, in particular the superior and posterior AC ligaments contribute to anterior/posterior stability of the AC joint, while the CC ligament complex (conoid and trapezoid) conveys superior/inferior stability. However, when the AC ligaments are ruptured, the conoid and trapezoid ligaments function to resist anterior and posterior forces, respectively. This highlights the importance of the CC ligament complex with regards to AC joint biomechanics and preventing instability not just vertically, but in the horizontal plane as well [19].

The trapezoid ligament attaches anterolaterally onto the distal clavicle, with the broader, robust conoid ligament attaching in a more posteromedial position. Biomechanical analysis has demonstrated the conoid and trapezoid tuberosities as distinct bony landmarks demarcating the anatomic relationship between the distal clavicle and coracoclavicular ligaments [18]. Rios et al. demonstrated that the distance from the distal edge of the clavicle to the medial aspect of the conoid tuberosity in male and female specimens was 47.2 ± 4.6 mm and 42.8 ± 5.6 mm, respectively. The distance to the trapezoid tuberosity was 25.4 ± 3.7 mm in males, and 22.9 ± 3.7 mm in females [18]. The distinct attachment sites of the conoid and trapezoid ligaments provide their inherent stability, and anatomical reconstruction techniques aim to re-establish this native anatomical relationship [1, 17–19].

### Patient selection

Low grade injuries such as type I-II according to Rockwood should be treated non-operatively [7], as current conservative management strategies show favourable outcomes, higher return to activity and less complications rates [1, 2]. In contrast, in high-grade injuries such as types IV-VI according to Rockwood surgery should be recommended [1, 2, 20]. However, to date, controversy arises if operative treatment is superior to conservative treatment, especially in the challenging grade III injury population. To date, clinical studies failed to show significant advantage for surgical interventions [11]. Functional impairment due to scapular dyskinesia or chronic pain remains has been showed to be highly concerning following non-operative treatment. Some studies indicate that operative treatment may result in less pain and better endurance, especially during overhead work [21, 22], as well as improved patient satisfaction in both short- and long-term follow-up studies [12, 23].

However, according to a meta-analysis, no differences between operative and nonoperative treatment have been observed in terms of shoulder strength, pain relief, throwing ability, or development of osteoarthritis (OA) [12]. In contrast, longer sick leave and better cosmesis may occur with operative treatment [12]. While operative treatment can address clavicular displacement and restore the radiographic alignment in grade III injuries, it has also been shown to improve subjective and objective outcome measures [24]. A survey from McFarland and colleagues conducted in professional throwing athletes showed that complete pain relief and return to ‘normal’ was achieved more predictably with operative treatment compared to nonoperative treatment (92% vs. 80%) [25]. Additionally, Cardone and Brown reported more satisfactory outcomes and a trend toward earlier return to Australian football following operative treatment [26].

Finally, the highly variable severity of type III injuries recently led to a consensus statement published by the International Society of Arthroscopy, Knee Surgery and Orthopaedic Sports Medicine (ISAKOS) to further subdivide type III injuries, in order to more accurately identify patients who may benefit from surgery [2]: type IIIA injuries are considered stable without overriding of the clavicle on the cross-body adduction view and without significant scapular dysfunction, whereas type IIIB injuries present with horizontal instability and therapy-resistant scapular dysfunction. Accordingly, persistent scapular dysfunction remains a major concern following AC joint injuries. The AC and CC ligaments have been noted to ensure a physiological motion of the scapula [27, 28], with injuries to those ligaments may result in a more protracted and internally rotated position of the scapula, consequently leading to motion deficits and shoulder pain [29]. Interestingly, Gumina and colleagues demonstrated that in patients with chronic type III injuries, scapular dyskinesis is present in 70.6% of cases, of whom 58.3% demonstrated a SICK scapula syndrome (scapular malposition, inferior medial scapular winging, coracoid tenderness, and scapular dyskinesis), which is associated with inferior shoulder function [30]. Unfortunately, 20% of these patients still present with scapular dysfunction, even after successful completion of conservative treatment [31]. Thus, delayed surgical intervention should be indicated in patients present with persistent pain, recurrent instability or severe scapulothoracic dyskinesia after failing a trail of conservative treatment for at least 3 to 6 weeks [2].

.

### History, physical examination, and diagnostic imaging

A detailed physical examination and accurate radiographic imaging are key for correct classification of the injury [2], including examination of glenohumeral joint, sternoclavicular joint, cervical spine and ipsilateral upper extremity along with a complete neurovascular exam to rule out concomitant injuries. Intraarticular comorbidities such as lesions of the long head of the biceps tendon or SLAP lesions (superior labral anterior posterior lesions) have been reported to occur in up to 18% of high grade AC joint dislocations [32], making these injuries a possible intra-articular pain source. Depending on the severity of the injury, ecchymosis and AC joint deformity may occur. In the absence of obvious AC joint deformity, tenderness to direct palpation over the AC joint and a painful cross body adduction test may indicate an injury of the AC joint. If needed, relief of symptoms by injection of local anaesthetic into the AC joint may be performed, however, this approach should be considered as an indirect and less common way of confirming the diagnosis.

Once AC joint injury has been confirmed, surgeons should focus on testing vertical displacement, horizontal and rotational instability. Horizontal stability of the AC joint is assessed by moving the clavicle in an anterior to posterior direction while stabilizing the acromion. Even though slight horizontal instability may not significantly influence clinical outcomes, physical examination should detect if present (chronic) horizontal or rotational instability may result in scapula dysfunction [33], or more importantly, in pain. Assessment of scapulothoracic motion is essential, as proper function of the AC joint is critical for correct scapulothoracic rhythm. Thus, in chronic AC joint injuries, the scapula may lack anterior strut resulting in excessive scapular internal rotation with anterior tilt [2], which may lead to persisting rotational and horizontal instability resulting in chronic pain. At this point, a detailed radiological evaluation using feasible and precise methods is required. However, a huge range of radiographic techniques without a clear standardized radiographic protocol have been described in current literature [34]. These radiographic techniques include: Bilateral Zanca view, bilateral panoramic view, (dynamic) axillary view and stress imaging.

### Radiographic assessment of vertical instability

**Fig. 1:**
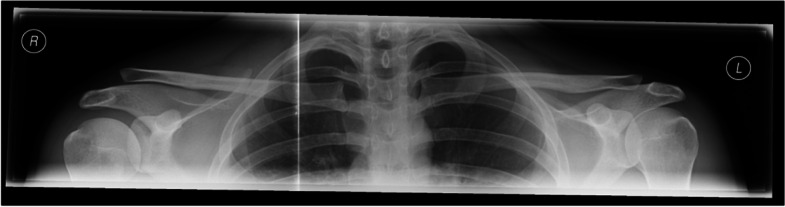
Preoperative bilateral panoramic view allowing for direct correlation of the CC-distance to the uninjured contralateral AC joint

**Fig. 2:**
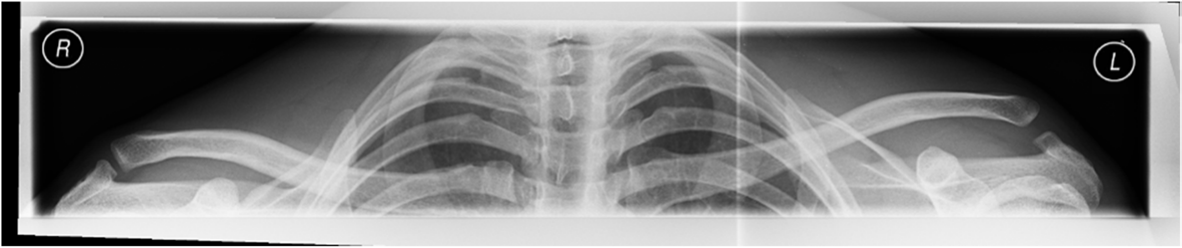
Preoperative bilateral panoramic view of a patient with Rockwood IIIB instability (Left side)

Vertical instability can be diagnosed with high inter- and intra-observer reliability in a bilateral panoramic view by measuring the coracoclavicular (CC) distance]. Bilateral views allow direct correlation of the CC-distance to the uninjured contralateral AC joint (Figs. 1 and 2) [35, 36].

### Assessment of horizontal and rotational instability

**Fig. 3:**
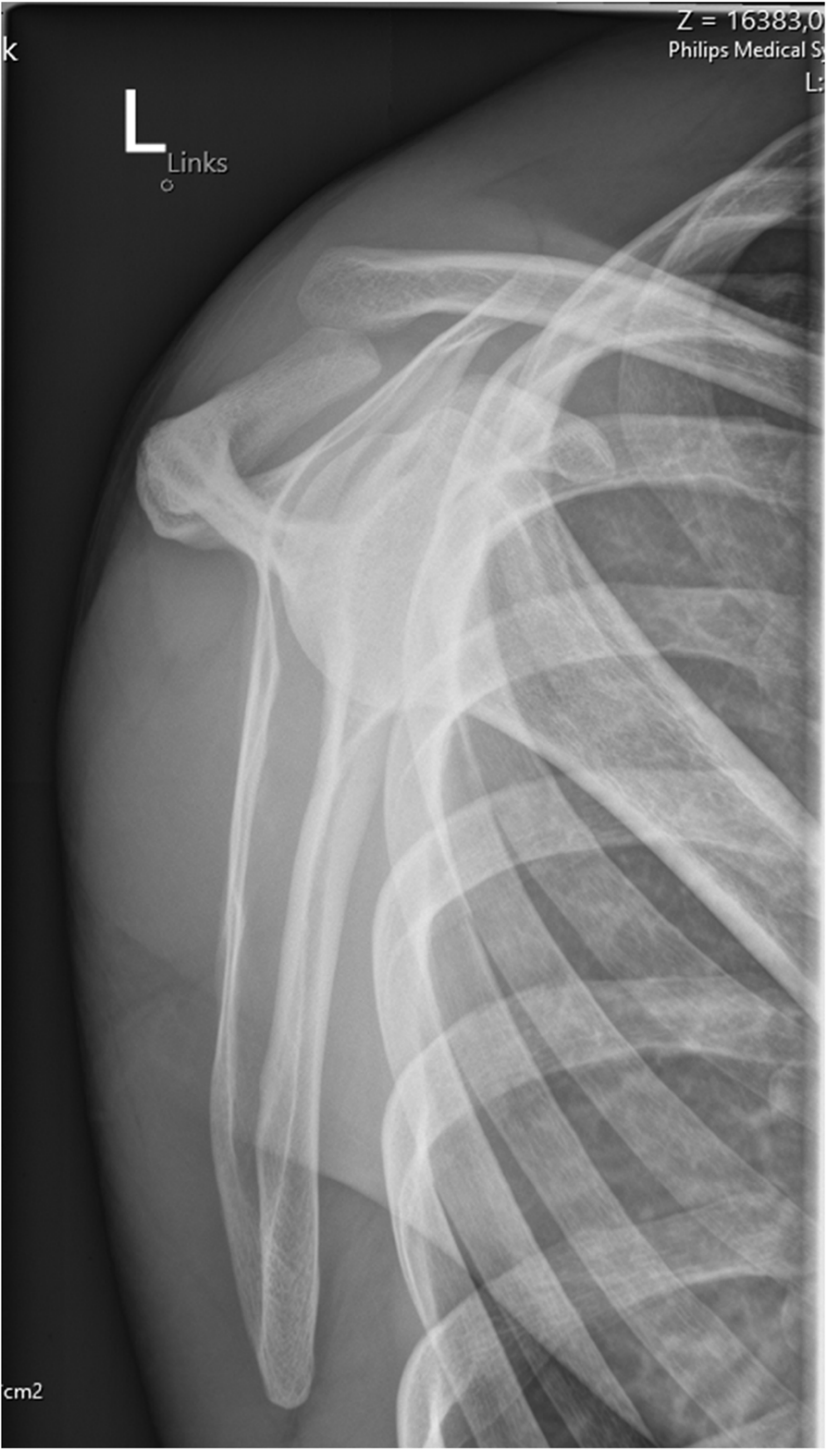
Preoperative modified y-view (Alexander view) allowing for visualization of dynamic horizontal instability (overriding of the lateral clavicle)

**Fig. 4:**
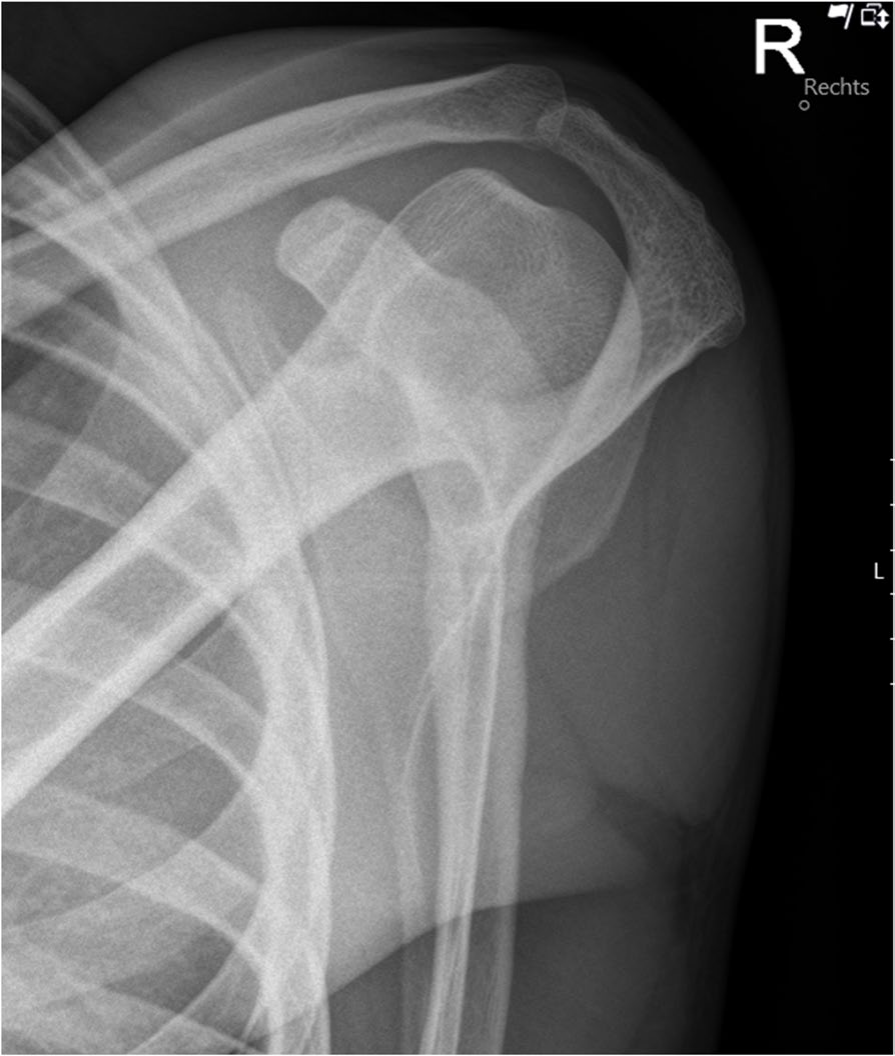
Preoperative modified y-view (Alexander view) of an intact AC joint without overriding of the lateral clavicle

Accurate assessment of horizontal instability has been shown to be one of the most important steps in AC joint treatment recommendations [1]. However, correct and reliable diagnosis is often difficult with heterogeneous inter- and intra-observer reliability being reported [34]. Literature suggests bilateral Alexander (modified y-view; Figs. 3 and 4) views [37] for quantifying dynamic horizontal instability, especially in patients with grade IIIB instability [38], however, there remains a lack of evidence in the advantage of this radiographic view [34]. In contrast, static horizontal instability (Rockwood type IV) may be best seen on axillary views Additionally, Karagyris et al. recently proposed the acromial center line to dorsal clavicle (AC-DC) distance to define watershed cases (i.e. IIIA/IIIB/IV) [39], while Zumstein et al. recommended the glenoid centre line to posterior clavicle (GC-PC) for assessing horizontal instability [40].

### Magnetic resonance imaging (MRI) and computed tomography (CT)

**Fig. 5:**
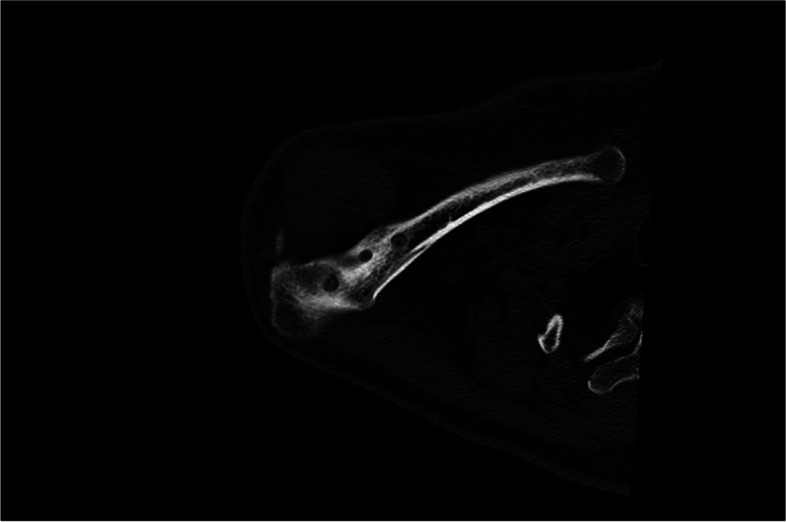
Computed tomography allowing for adequate visualization of bony imparities such as clavicular bone tunnel position, tunnel width or fractures

**Fig. 6:**
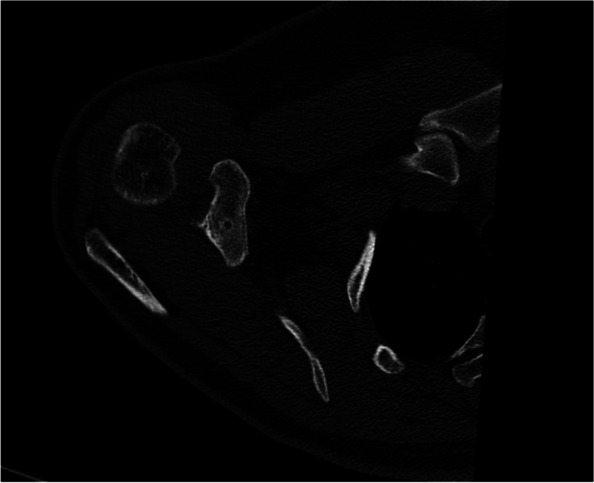
Computed tomography allowing for adequate visualization of bony imparities such as coracoid bone tunnel position, tunnel width or fractures

In order to complete an exact examination, MRI scans may be useful for detecting concomitant injuries of the glenohumeral joint, including SLAP lesions or rotator cuff tears as they occur in up to 20% of the cases [32, 41]. In chronic cases, CT scans may be helpful for a detailed visualization of osseous structures. In case of revision surgery, CT diagnosis is essential to detect insufficient fixation, technical failures (tunnel widening, tunnel position; Figs. 5 and 6) or clavicular and/or coracoid fractures.

## Choosing the optimal surgical technique

### Open procedures

#### Anatomic Coracoclavicular ligament reconstruction (ACCR)

Historically, an anatomic and biologic solution for CC ligament reconstruction was eloquently described in 1928 by Bunnell, who incorporated a fascial graft weave that offered stability between the clavicle and the scapula at both sites [42]. Since then, anatomic and biologic approaches for AC joint reconstruction have gained popularity [43], as reliable restoration of function and comfort is seen as dependent on the durable restoration of anatomic parameters, including the congruency and stability of the AC joint. A first clinical trial has been described by Jones and colleagues [44], followed by several biomechanical investigations demonstrating that anatomic coracoclavicular ligament reconstruction (ACCR) using autograft or allograft tendon to replicate the CC ligaments at their anatomic location are more effective in mimicking the properties of the native CC ligaments compared to ligament transfers [45–48].

#### Biomechanical investigations

When compared to thee (modified) Weaver-Dunn procedure, nonanatomic allograft, anatomic suture, and graft-rope techniques, ACCR demonstrates superior load-to-failure characteristics [45, 46, 49]. In addition, anatomic reconstruction of the CC ligaments best restores the biomechanical properties of the native CC ligaments [45, 46], reproducing peak loads equivalent to that of the native CC ligaments, however, with lower stiffness [50, 51]. Further, Costic showed that anatomic reconstruction with a semitendinosus tendon failed to demonstrate significant graft elongation during cyclic loading [47].

Optimal tunnel placement in the clavicle is of high importance to achieve optimal strength and reduce potential risk of failures [43]. Geaney et al. showed that tunnel placement in the clavicle corresponding to the attachment of the CC ligaments has the highest bone marrow density (BMD), and correlates to higher loads to failure experimentally [52]. To reduce persistent horizontal and rotational instability of the AC joint following reconstruction [14, 53], the AC capsule should be reconstructed [14, 51, 54]. Voss and colleagues showed, that the posterior-medial acromion closest to the AC joint reveals the highest BMD with increasing density from lateral to medial; thus, fixation at this location might be favorable [55], with tunnels placed at the acromion within the “safe zone” (i.e., within the anterior half of the acromion) to not affect the load-to-failure at the acromion [56]. However, as persistent rotational instability remains a concern and may play a significant role in AC joint reconstruction failure [57, 58], future research is warranted in this area.

#### Surgical outcomes and complications

Open anatomic techniques generally yield in good and satisfactory outcomes [43, 59]. Muench et al. reported on 43 patients undergoing ACCR for acute and chronic type III and V AC joint injuries with 81% of patients reaching the substantial clinical benefit (SCB) after surgical reconstruction [20]. Similar, good outcomes can be expected in revision cases using the ACCR technique [60].

### Coracoacromial ligament and tendon transfer

Coracoacromial ligament (CAL) transfer as an operative approach for AC joint injuries has a long history and dates back to 1917 [43], when Cadenet treated dislocations and fractures of “the outer end of the clavicle” using his technique [13]. In the early 1950s, Neviaser advocated [61] and Weaver and Dunn (WD) finally modified the technique and used it for both acute and chronic type III injuries [62]. Since then, a diversity of modifications has been proposed, all using the CAL transfer or WD technique in the same or a similar manner [63–69]. Accordingly, the CAL is transferred to the clavicle with or without an accompanying fragment of bone and may be enhanced by supplementary fixation. In 1965, Dewar et al. first described tendon transfers as a treatment for AC joint injuries [70], by performing an osteotomy of the tip of the coracoid process, including the coracobrachialis and the short head of the biceps tendon and attaching it to the clavicle [71–73].

### Biomechanical investigations

When compared to intact CC ligaments, the CAL transposition as used in the Weaver-Dunn reconstruction is biomechanically significantly weaker and lax [48, 74–76]. Mazzocca and colleagues showed, that the (modified) Weaver-Dunn procedure failed to reproduce the load-to-failure durability of the intact AC and CC ligament complex [43, 46]. In contrast, LaPrade et al. demonstrated that motion at the AC joint may be restored to near-normal values,when the Weaver-Dunn reconstruction is combined with coracoid transclavicular cerclage [77]. Similar, Lee et al. concluded that for sufficient resistance to AC joint motion, surgeon’s should consider using a biological graft to further enhance the Weaver-Dunn reconstruction [78]. However, when comparing the Weaver-Dunn reconstruction to more “anatomic” reconstructions, (modified) Weaver-Dunn reconstructions are considered biomechanically inferior [49, 79, 80].

Tendon transfers have also been investigated biomechanically. Sloan and colleagues recognized, that the strength of the lateral half of the conjoined tendon (265 N) was inferior to the intact ligaments, but similar to that of the CAL (246 N) [81]. Of interest, Wellmann et al. advocated the use of the pectoralis minor tendon to prevent the complications that may arise from the CAL transfer, as the pectoralis major showed similar biomechanical properties compared to the CAL [82].

### Surgical outcomes and complications

Using the CAL transfer or modified Weaver-Dunn technique may yield in satisfactory results [62, 66, 68, 83], however, to date there is only low-level evidence to support the clinical use of CAL transfer [84]. Similarly, mixed results have been reported for the treatment of type III injuries with transposition of the tip of the coracoid process [71, 85, 86], thus, this procedure may not be indicated in this patient cohort [43, 85].

### AC joint reconstruction with hook plates

In 1976, Balser advocated the use of a hook plate in the treatment of AC joint dislocations for the first time [87]. In the past decade, different hook plate designs have been presented, all based on the same principles: open reduction and internal fixation (ORIF) with a precontoured hooked plate affording rigid internal fixation and sparing the articular surfaces of the joint [43, 88–90]. Advantages of hook plates comprise the simultaneous stabilization of CC and AC ligaments, which is mostly afforded by scar tissue. Several modifications have been described to further enhance the construct, including direct CC ligament repair, suturing the capsuloligamentous complex, additional screw fixation, biological or artificial augmentation or ligament/tendon transfers [89, 91, 92]. However, implant removal may be needed between 4 and 24 weeks postoperatively [93], whereas some authors do not routinely remove the device [94].

### Biomechanical investigations

Patients undergoing hook plate fixation may demonstrate reduced internal rotation along with increased anterior translation (2 mm) of the clavicle with respect to the medial acromion, when compared to the native AC joint [43, 95]. Compared to TightRope and bone anchor systems, Nüchtern and colleagues demonstrated higher axial stiffness for the hook plate [96].

### Surgical outcomes and complications

The use of hook plates has become extensively widespread especially in Europe, with consistent high clinical outcomes being reported [43, 88, 90, 93, 94, 97, 98], along with poor correlations between clinical and radiographic results being observed [90, 92, 94]. In case of persistent anterior-posterior instability, clinical scores have been shown to be lower [90, 99], however, early return to work and sports remains common [97–99]. When comparing acute or delayed treatment, Ejam et al. found no significant differences in clinical outcomes [93]. Additionally, Gstettner et al. showed better radiographic and clinical outcomes in patients treated with the hook plate compared to nonoperative treatment [89]. Interestingly, Di Francesco and colleagues observed scarring/healing of the CC ligaments in 88% of patients on MRI [98]. Subsequent plate removal has been demonstrated to not lead to loss of reduction or poorer clinical outcomes [100].

However, Mah et al. questioned the efficacy of hook plate stabilization in the setting of acute, high-grade AC joint instability, as they found no difference in general health status between nonoperative and operative treatment [101].

### Arthroscopic assisted techniques

#### Suture augmentation and synthetic devices

Multiple kinds of suture augmentation and synthetic ligaments have been used for AC joint stabilization [43, 102–104], and can be passed extraosseous, intraosseous, and transosseous using drill holes in the clavicle, the coracoid, or in both. Advantages include less risk of neurovascular damage, less blood loss, shorter operative time, or no need for potential hardware removal [14, 105]. By passing nonabsorbable sutures through anatomic tunnels at the location of the conoid and trapezoid ligament footprints [106], optimal construct strength may be achieved. Additionally, CC ligament repair may be performed [105, 107].

**Fig. 7:**
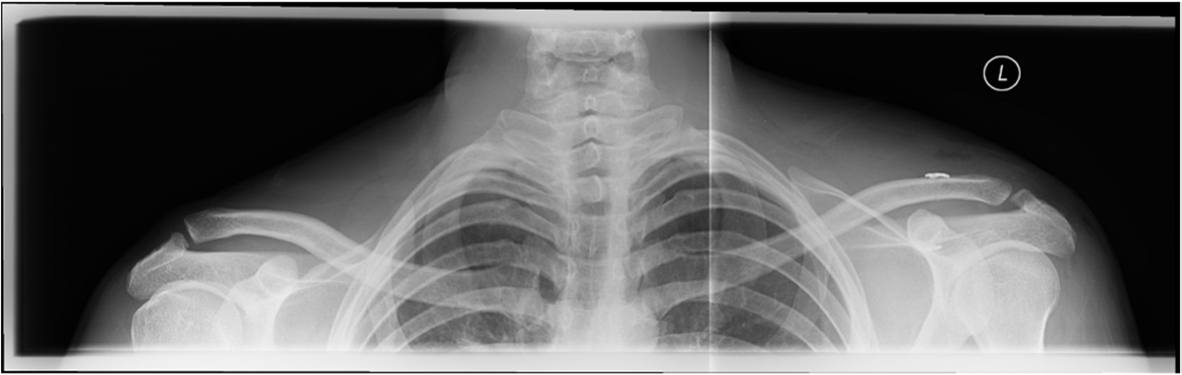
Postoperative bilateral panoramic view after stabilization of chronic Rockwood type IIIB AC joint instability using the arthroscopic-assisted hybrid technique

**Fig. 8:**
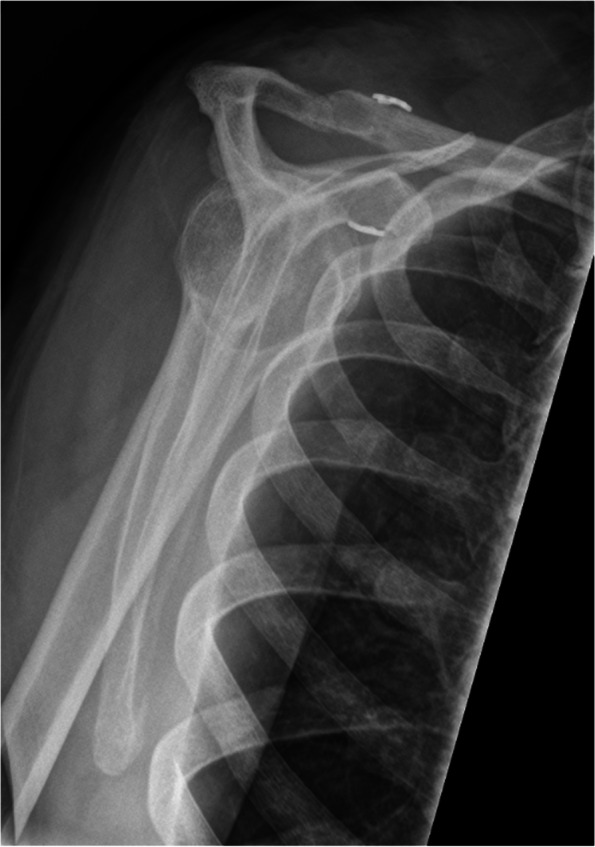
Postoperative y-view after stabilization of chronic Rockwood type IIIB AC joint instability using the arthroscopic-assisted hybrid technique

Additionally, newer constructs such as high-tensile sutures with endobutton fixation have been advocated over the past years [14, 38, 90, 108–110], (Figs. 7 and 8) with the first arthroscopic-assisted AC joint reconstruction dating back to 2002 [111, 112]. Especially in chronic or revision cases, biological augmentation may be required to support healing of the torn structures and preserve stability of the reconstruction [113]. The use for biologic grafts may not be dependent on the strength of the reconstruction, which can be comparably achieved with nonbiologic materials, but rather on the necessity of a biologic substrate [114].

#### Biomechanical investigations

Biomechanically, suture augmentation may act as a temporary internal brace, maintaining reduction of the AC joint until ligamentous healing is complete [14, 43, 115, 116]. Synthetic suture devices have failure loads comparable to the intact CC ligament (725 N) when passed around or through the clavicle [115]. Contrary, Martetschläger et al. demonstrated inferior biomechanical properties of braided polyethylene suture (PDS) sutures used to reconstruct the AC and CC ligaments [117]. Thus, PDS may be too weak to achieve vertical stability [117], however, it is considered biomechanically superior compared to direct coracoid suture anchor repair [114].

As adequate tunnel placement if of great importance for recreating native anatomy, various methods to pass synthetic sutures through the clavicle and/or coracoid, with or without biologic augmentation, and methods to secure sutures and/or grafts have been described. Especially in chronic or revision cases, biological augmentation may be required to support healing of the torn structures and preserve stability of the reconstruction [113]. Native-like biomechanical properties of double graft tendons using hybrid techniques, such as the combination of Tight Ropes and Fibertapes with tendon grafts, may be achieved [46, 47]. Similar biomechanical properties may be expected when comparing coracoid-side flip-button tendon graft with tendon looping around the coracoid and synthetic suture augmentation [118]. Passage via double tunnels in both the clavicle and the coracoid may result in a reconstruction close to the native state compared with a single tunnel in the coracoid [119], especially when using a transosseous TightRope (Arthrex Inc., Naples, FL, USA) method [120]. This observation contradicts findings from Beitzel et al., who showed no difference between one and two tunnels in the clavicle [79, 80]. Ferreira and colleagues demonstrated higher load-to-failure of the repair construct when placing the coracoid bone tunnel center-and-center or medial-and-center, respectively [121]. However, Coale et al. found significant risks when attempting restoration of the anatomic footprint of the conoid and trapezoid ligaments [122]. Recreating both anatomic ligament footprints may enhance significant risk of cortical breach and fracture [122, 123]. Further, tunnel size, especially in the clavicle, is of great importance, as larger bone tunnels may increase fracture risk [124, 125]. Placing a hamstring tendon graft through 6 mm tunnels significantly weakens the clavicle compared to a cortical button and suture placed through 2.4 mm tunnels [124].

Finally, repair of the AC capsule may result in improved horizontal and rotational stability [54, 126], as almost 80% of the horizontal stability is provided by an intact superior-posterior capsuloligamentous complex. ^28, 29, 50, 67, 7^ Thus, these structures should be addressed in chronic cases, as persisting horizontal instability may lead to chronic pain and limited shoulder function [17, 57, 58, 127, 128]. When using different types of synthetic material for horizontal AC joint stabilization, no differences in outcomes may be expected. However, biomechanically, a box-shaped configuration for AC joint capsule repair may be best suited for optimal repair and may be superior to a Figure of eight configuration [58].

#### Surgical outcomes and complications

Generally, when reconstructing the AC joint using synthetic devices and ligaments, favorable clinical outcomes and return to preinjury activity levels may be expected [43, 92, 105, 106]. At 2 years, a 83% survivorship using these techniques has been identified [15].

When using transclavicular nonabsorbable sutures secured to the coracoid with suture anchors, satisfactory clinical outcome scores may also be expected [129]. Additionally, transosseous sutures spanning the CC interval secured through endobutton fixation on the lateral clavicle lead to early clinical success rates of nearly 90% along with radiographic stability restoration [99, 109, 130]. Rosslenbroich et al. demonstrated that younger patients achieve higher outcome values [131]. When assessing AC joint capsule repair, Tauber et al. found that combined AC and CC Ligament reconstruction better restored horizonal stability and that patients undergoing combined reconstruction showed improved patient reported and radiographic outcomes [132]. An increased risk for osteoarthritis around the AC joint has not been described so far.

Although the efficacy of Ligastic, Ligament Augmentation and Reconstruction System (LARS) or double braided polyester devices has been confirmed [133], structural failures of this device leading to inferior clinical outcomes may occur.

## Discussion and therapeutic decision making

Based on the available evidence, firm conclusions are challenging regarding operative versus nonoperative treatment, the timing of surgery, open versus arthroscopic surgery, and choice of surgical procedure. Several studies demonstrated the lack of correlation between clinical outcomes and abnormal radiologic findings [3, 4, 6, 14]. As there is currently limited level I evidence, treatment considerations are mostly based on surgeon-specific factors such as experience and patient-specific factors such as age or functional demands,

In the majority of cases, nonoperative treatment of acute low-grade injuries is seen as appropriate, despite the risk of continuous pain and future development of chronic AC joint instability. High-grade injuries are typically managed operatively due to the loss of stability of the shoulder girdle and subsequent scapulothoracic imbalance [1–4, 6, 11, 14, 23, 24, 134].

**Fig. 9:**
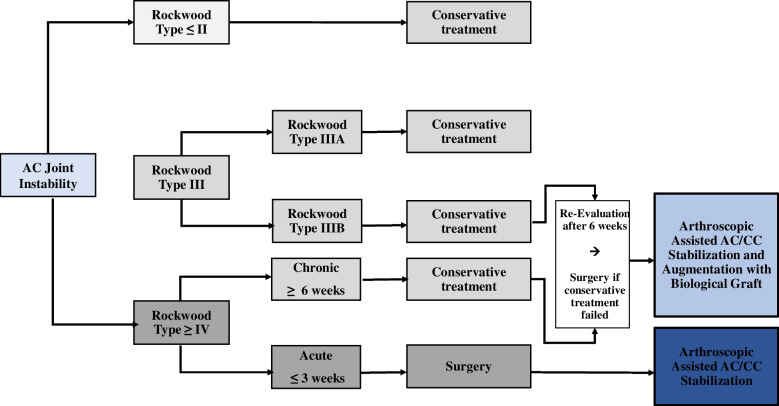
Non-operative or operative treatment based on the author’s therapeutic decision making

The treatment algorithm (Fig. 9) proposed in this review is focused on specific and clinically relevant considerations based on the available literature. The authors of this paper developed a therapeutic decision making based on information’s obtained from recent clinical and biomechanical studies. Interestingly, within the past decade, an exponential increase in arthroscopically assisted techniques has been published, which demonstrates the raised importance of combined procedures. In addition, arthroscopic approaches may decrease the risk of serious soft tissue infection [135].

As the AC joint ligaments lose their potential to heal after 3 weeks following injury [68, 136], less than 3 weeks has been established as a cut-off for acute injuries [2, 137]. Thus, patients presenting with acute high-grade AC joint injuries (e.g. types IV, V, VI) should be indicated for operative treatment [2]. However, when an acute AC joint dislocation is graded as type III, an initial trial of conservative treatment may be indicated, thus making the definition “acute” challenging. Patients with a stable AC joint without overriding of the clavicle and without significant scapular dysfunction (Type IIIA) may benefit from a more conservative, nonoperative approach in contrast to patients with overriding of the clavicle and therapy-resistant scapular dysfunction (Type IIIB). If these patients fail to respond to conservative measures, an anatomic AC joint reconstruction using a hybrid technique should be considered. Besides, acute superior acromioclavicular ligament complex injuries were shown to follow distinct tear patterns, including clavicular-sided tears, oblique tears, midportion tears and acromial-sided tears. It has been shown that type-specific operative repair of acute acromioclavicular ligament complex tears might promote biological healing, consequently, lower rates of horizontal AC joint instability may occur [18], .as the integrity of the CC and AC ligaments has an impact on clinical and radiographic parameters [19].

In chronic AC joint injuries, surgery is indicated after failed nonoperative treatment of 3 to 6 months. Anatomic AC joint reconstruction techniques along with biologic augmentation (e.g. Hybrid techniques, combining Tight Rope) should be considered for chronic high-grade instabilities, accounting for the lack of intrinsic healing and scar-forming potential of the ligamentous tissue in the chronic setting. This approach has been demonstrated to provide better results regarding anterior and superior translation of the clavicle.^412^ Additionally, recent studies demonstrated native-like biomechanical properties of double graft tendons using hybrid techniques, such as the combination of Tight Ropes and Fibertapes with tendon grafts. However, as complication rates have been reported to be as high as 30% of cases, focus is placed more on reducing the size and number of bone tunnels during arthroscopically assisted stabilization techniques [15, 138]. Recent studies have shown the risk of postoperative fractures (clavicular and/or coracoid) to be related to the number and size of bone tunnels [56, 139, 140]. Therefore, reducing both variables may be of benefit in the setting of chronic ACJ stabilization. Focus should also be placed on restoring physiological horizontal and vertical ACJ stability. As the AC capsule and deltotrapezoidal fascia are significantly contributing to horizontal sand rotational stability, augmentation of the AC capsule is required.

### Limitations

The concepts and careful conclusions presented in this review are largely limited to the lack of high-level studies found in the literature. The large heterogeneity of studies made direct comparisons as well as drawing of definite conclusions regarding treatments difficult. However, the authors aimed to customize the suggested algorithm to the available clinical and biomechanical evidence.

### Future perspectives

The simple fact that over 150 different operative treatment strategies for AC joint injuries have been developed since 1917 emphasizes that the vast majority of the current techniques does not allow for complete restoration of native physiological and biomechanical AC joint properties. In the past decade, some authors advocated the importance of recreating the AC ligaments and capsule, as chronic horizontal and rotational instability may lead to chronic pain, and clinical failures [57, 58, 128]. Future clinical and biomechanical investigations should focus on addressing horizontal and rotational instability, as it remains a common challenge for shoulder surgeons. In addition, surgical techniques should only be classified as “anatomic”, if they “anatomically” reproduce the conoid and trapezoid ligaments.

Of interest, future studies may investigate on clinical and biomechanical outcomes on the newest arthroscopic-assisted technique, the knotless Tight Rope technique (2nd generation). By using this technique, less abrasive wear or shield stress (especially in bone tunnels) may be expected due to the knotless technique, which may reduce subsequent clinical failures. However, as for every new technique released, its advantages and superiority as well as its biomechanical properties still have to be demonstrated.

To this, despite advances in surgical techniques with additionally addressing the AC joint capsule using cerclages or the excess graft in order to improve horizontal stability, these approaches may not be able to adequately ensure rotational stability, which may subsequently lead to the observed postoperative failures [57, 58, 128]. Finally, a consent on optimal timing of surgery including the definition of “acute” and “chronic” has to be taken in future trials.

## Conclusion

Finding the right patient, establishing the correct diagnosis, and implementing the appropriate surgical technique remains a major challenge for shoulder surgeons. In the past decade, a trend towards arthroscopic assisted techniques has been noted. However, complication and clinical failure rates remain high, which may be a result of technical failures or persistent horizontal and rotational instability. Thus, future research should focus on addressing horizontal and rotational instability, to restore native physiological and biomechanical properties of the AC joint.

## Data Availability

All authors had unrestricted access to all the data of this study. Raw data can be requested from the corresponding author.

## Abbreviations

AC: Acromioclavicular
CC: Corococlavicular
ACJ: Acromioclavicular joint
SICK scapula syndrome: Scapular malposition, inferior medial scapular winging, coracoid tenderness, and scapular dyskinesis
SLAP lesions: Superior labral anterior posterior lesions
MRI: Magnetic Resonance Imaging
CT: Computed Tomography
ACCR: Anatomic Coracoclavicular Ligament Reconstruction
BMD: Bone marrow density
SCB: Substantial clinical benefit
CAL: Coracoacromial ligament
WD: Weaver and Dunn
N: Newton
ORIF: Open reduction and internal fixation
LARS: Ligastic, Ligament Augmentation and Reconstruction System
